# Archaea and Bacteria Acclimate to High Total Ammonia in a Methanogenic Reactor Treating Swine Waste

**DOI:** 10.1155/2016/4089684

**Published:** 2016-09-20

**Authors:** Sofia Esquivel-Elizondo, Prathap Parameswaran, Anca G. Delgado, Juan Maldonado, Bruce E. Rittmann, Rosa Krajmalnik-Brown

**Affiliations:** ^1^Swette Center for Environmental Biotechnology, The Biodesign Institute, Arizona State University, P.O. Box 875701, Tempe, AZ 85287-5701, USA; ^2^School of Sustainable Engineering and the Built Environment, Arizona State University, Tempe, AZ, USA; ^3^Department of Civil Engineering, Kansas State University, 2118 Fiedler Hall, Manhattan, KS 66506, USA

## Abstract

Inhibition by ammonium at concentrations above 1000 mgN/L is known to harm the methanogenesis phase of anaerobic digestion. We anaerobically digested swine waste and achieved steady state COD-removal efficiency of around 52% with no fatty-acid or H_2_ accumulation. As the anaerobic microbial community adapted to the gradual increase of total ammonia-N (NH_3_-N) from 890 ± 295 to 2040 ± 30 mg/L, the Bacterial and Archaeal communities became less diverse. Phylotypes most closely related to hydrogenotrophic* Methanoculleus *(36.4%) and* Methanobrevibacter* (11.6%), along with acetoclastic* Methanosaeta* (29.3%), became the most abundant Archaeal sequences during acclimation. This was accompanied by a sharp increase in the relative abundances of phylotypes most closely related to acetogens and fatty-acid producers (*Clostridium*,* Coprococcus*, and* Sphaerochaeta*) and syntrophic fatty-acid Bacteria (*Syntrophomonas*,* Clostridium*, Clostridiaceae species, and Cloacamonaceae species) that have metabolic capabilities for butyrate and propionate fermentation, as well as for reverse acetogenesis. Our results provide evidence countering a prevailing theory that acetoclastic methanogens are selectively inhibited when the total ammonia-N concentration is greater than ~1000 mgN/L. Instead, acetoclastic and hydrogenotrophic methanogens coexisted in the presence of total ammonia-N of ~2000 mgN/L by establishing syntrophic relationships with fatty-acid fermenters, as well as homoacetogens able to carry out forward and reverse acetogenesis.

## 1. Introduction

Animal wastes contribute more than half of the biomass-based wastes generated in the United States [1, 2]. The organic carbon in animal wastes could be a major source of renewable energy if it were captured as methane gas. Many animal wastes, including swine waste, also are rich in organic nitrogen (N) due to the high protein content in the animals' diet. Anaerobic hydrolysis and fermentation convert the organic N into ammonia-N (NH_3_-N). While typical NH_3_-N (i.e., unionized NH_3_ and NH_4_^+^) concentrations in anaerobic digesters treating domestic wastewater sludge are 650–1100 mg L^−1^ [3], concentrations in swine manure are as high as 8000 mg L^−1^ [4–6]. A challenge arises for treating these wastes (and ultimately capturing the organic carbon as energy source) as NH_3_-N above 1000 mg L^−1^ is toxic to many groups of microorganisms [7, 8], including methanogenic Archaea [9, 10].

In anaerobic systems without inhibition by NH_3_-N, organic acids produced from acidogenesis are fermented to acetate and H_2_, and the typical distribution of the electron flow to methane is 67% through acetate and 33% through H_2_ [11, 12]. Correspondingly, acetoclastic methanogens usually predominate in anaerobic digesters with <1000 mg NH_3_-N L^−1^ [13, 14]. In contrast, the vast majority of studies on methanogenesis from swine waste report that the dominant methanogenesis pathway switches from acetoclastic to hydrogenotrophic. Several studies [15–19] reported that the methane production in anaerobic reactors treating wastes with high NH_3_-N occurs mainly via hydrogenotrophic methanogenesis, since acetoclastic methanogens are inhibited and washed out. The loss of acetoclastic methanogens in the bioreactors raises questions about the fate of acetate generated by fermentation. It was previously postulated that the loss of acetoclastic methanogens was compensated by syntrophic “acetate oxidation” to CO_2_ and H_2_ (more accurately termed reverse acetogenesis) coupled with hydrogenotrophic methanogenesis [20]. Specifically, acetate generated by fermentation is converted into H_2_ and CO_2_ by reverse acetogenesis, and the H_2_ and CO_2_ are utilized by hydrogenotrophic methanogenesis. The reactions involved in the syntrophy of reverse acetogenesis coupled with H_2 _conversion into methane with their corresponding Δ*G*°′ are illustrated in Table 1, equations (1a) and (2), respectively. Reverse acetogenesis might not be the only mechanism that allows acetate conversion into methane when treating high-ammonium wastes. Methanogenesis is possible if some acetoclastic methanogens are able to adapt to high-ammonium concentrations and avoid being washed out. In fact, Westerholm et al. detected acetoclastic methanogens in methanogenic reactors operating at increasing NH_3_-N concentrations (from 800 to 6900 mg L^−1^) [21]. Thus, an acclimation period may be crucial for developing a microbial community that has acetoclastic methanogens capable of tolerating high NH_3_-N.

Another key aspect of microbial community acclimation is the scavenging of H_2_ produced in fermentation and reverse acetogenesis. It is well known that fermentation of propionate (equation (1b) in Table 1) and butyrate (equation (1c)) is coupled with hydrogenotrophic methanogenesis [22–24]. The highly endergonic nature of these fatty-acid fermentation instances means that they can occur only at very low partial pressures of H_2_ (<10^−4^ atm), which requires a tight syntrophic partnership with a H_2_ scavenger, such as a hydrogenotrophic methanogen, to maintain a negative Δ*G*°′ for fermentation to proceed [25].

The microbial ecology of anaerobic reactors treating high NH_3_-N wastes has received limited attention [15, 26–28], and the possible syntrophies among different Archaea, fermenting Bacteria, and homoacetogens are yet poorly understood. High-throughput sequencing in combination with statistical analysis can illuminate microbial dynamics with high NH_3_-N concentrations by identifying key Archaea and Bacteria involved in syntrophic fatty-acid conversion into methane. In this study, we used high-throughput sequencing, parametric correlation, and qPCR (quantitative polymerase chain reaction) to analyze shifts in the Archaeal and Bacterial communities during the startup phase (first 105 days) of an anaerobic digester successfully treating swine manure to generate methane and without significant accumulation of acetate or H_2_. Contrary to previous explanations of the effects of NH_3_-N concentration higher than 1000 mg L^−1^, acetoclastic methanogens played a major role in methane production. Our results point to syntrophies that involved acetoclastic methanogens, hydrogenotrophic methanogens, homoacetogens, and other syntrophic acid-fermenting Bacteria developed during the startup of a methanogenic bioreactor able to function well with NH_3_-N greater than ~2000 mg NH_3_-N L^−1^.

## 2. Materials and Methods

### 2.1. Anaerobic Bioreactor Setup and Operation

The bioreactor consisted of a 1-L glass vessel continuously stirred at 170 rpm and operated at 37°C and at a pH of 6.9–7.6. The reactor was inoculated with 1 : 2 volume ratio of anaerobic digested sludge to swine waste to ensure that the concentration of NH_3_-N was below 1100 mg N L^−1^. The anaerobic digester sludge was obtained from the Northwest Wastewater Reclamation Plant (Mesa, Arizona, USA) and the swine waste from Hormel Foods (Snowflake, Arizona, USA).

The bioreactor was operated in batch mode for 35 days, during which time methane production reached a plateau. Afterwards, the operation was switched to semicontinuous mode by daily removing and adding 33 mL of swine waste feed with a sterile syringe under vigorous N_2_ sparging. The hydraulic retention time (HRT), equal to the solids retention time (SRT), was 35 days (denoted as “cycle” in the manuscript figures). The bioreactor was operated semicontinuously for 105 days.

### 2.2. Chemical Analyses

An array of chemical analyses was employed to characterize the input swine waste and to monitor the performance of the anaerobic reactor through liquid samples taken from the reactor's homogenized contents. Total and soluble chemical oxygen demand (TCOD and SCOD), soluble total nitrogen (TN), and NH_3_-N were assayed with HACH® kits using spectrophotometer absorbance at wavelengths of 620, 410, and 655 nm, respectively. Soluble concentrations were measured after filtering the sample through a 0.45 *μ*m membrane filter.

Gas samples (200 *μ*L) were withdrawn from the bioreactor headspace using a 500 *μ*L gas-tight syringe (SGE, Switzerland) to quantify methane (CH_4_) and hydrogen (H_2_) concentrations using a gas chromatograph (GC 2010, Shimadzu) equipped with a thermal conductivity detector and a packed column (ShinCarbon ST 100/120 mesh, Restek Corporation). N_2_ was the carrier gas supplied at a constant pressure of 405 kPa and a constant flow rate of 10 mL min^−1^, and the temperature conditions for injection, column, and detector were 110, 140, and 160°C, respectively.

### 2.3. DNA Extraction

Four biomass samples were obtained from the inoculum and the effluent of the anaerobic bioreactor at the end of the batch operation and at the end of the first and second cycles of the semicontinuous operation, when the system was at steady state based on the COD removed as CH_4_. Effluent samples were pelleted using an Eppendorf microcentrifuge 5810R (Hauppauge, NY) at 13,200 rpm. DNA was extracted from 0.25 g (wet weight) of two pellets per sampling point using the MOBIO PowerSoil® DNA extraction kit (Carlsbad, CA). DNA from duplicate pellets was merged for sequencing.

### 2.4. High-Throughput Microbial Community Analysis

To determine the structure of the Bacterial community during startup of the semicontinuous reactor, we sequenced DNA using the Illumina MiSeq platform at University of Minnesota Genomics Center (http://www.health.umn.edu/research/resources-researchers/genomics-center). Bacterial primers used were V4F (5′-GTGCCAGCMGCCGCGGTAA-3′) and V6R (5′-ACAGCCATGCANCACCT-3′), which amplify the V4–V6 hypervariable regions of the 16S rRNA gene. The reads were paired-end, and each end of the DNA fragment consisted of 300 bp (2 × 300 bp). Before processing the reads using the QIIME 1.8.0 pipeline [29], we paired forward and reverse sequences using PANDASeq [30]. The average length of reads after overlap was 551 bp.

The Archaeal community was sequenced at MR DNA (http://www.mrdnalab.com/, Shallowater, TX, USA) on an Illumina MiSeq following the manufacturer's guidelines. 16S rRNA primers 349F and 806R, with barcode on the forward primer, were used to amplify the V3 and V4 hypervariable region of this highly conserved gene [31]. The reads were paired-end, and each end of the DNA fragment consisted of 300 bp (2 × 300 bp). The average length of reads after overlap was 449 bp. Bacterial and Archaeal raw sequences were submitted to NCBI Sequence Read Archive and are available under the following accession numbers: SAMN04481086–SAMN04481094.

Sequences with at least one of the following characteristics were omitted for the downstream analysis: being shorter than 200 bps, with quality score of 25 or below, with any primer or barcode mismatches, and with more than 6 homopolymers. From the sequences that passed the quality filtering, OTUs were picked based on 97% sequence similarity using the UCLUST algorithm [32]. The most abundant sequence of each cluster was picked as the representative sequence. Sequences were aligned using the PyNAST method [33] and filtered to remove gaps and Chimeras (using ChimeraSlayer [34]). The UCLUST algorithm [32] was used to assign taxonomy to the most abundant sequence of each OTU by comparing the most abundant sequence of each OUT to the Greengenes database [35]. OTU tables (1 each for Bacterial and Archaeal sequences) were generated from the representative sequences excluding Chimeras. OTUs with single sequences (singletons) were removed from the OTU tables. To avoid biases that occur when sampling various species in a community, OTU tables were subsampled (rarefied) using the pseudorandom number generator (PRNG) NumPy, an implementation of the Mersenne PRNG [36]. Final OTUs numbers were 3275 for Bacteria and 776 for Archaea. The numbers of high-quality reads per sample in the Bacteria and Archaea analysis were 25,000 and 90,000 sequences, respectively, on average.

### 2.5. Sample Diversity

We calculated sample species diversity (*alpha* diversity) by estimating PD- (Phylogenetic Distance-) whole-tree and observed-species metrics using the QIIME 1.8.0 pipeline [29]. For this, we performed multiple subsamplings (rarefactions) of the OTU table (Bacteria and Archaea separately) at a depth of 100 sequences in 10 replicates, and we analyzed rarefaction measures in which the sequence numbers per sample were equal (18,000 sequences per sample for Bacteria and 32,000 for Archaea).

### 2.6. Quantitative Real-Time PCR (qPCR)

We performed qPCR in 20 *μ*L reactions, each containing 6 *μ*L PCR grade water, 0.04 *μ*L TAQMAN probe (200 nM), 1 *μ*L each of forward and reverse primers (500 nM), 10 *μ*L TAQ PCR SuperMix (1X) or SYBR green mix (1X), and 2 *μ*L template normalized DNA to 10 ng/*μ*L (130 nM). Using TAQMAN assays, we used the qPCR primers and conditions described previously [37, 38] for the amplification of the 16S rRNA gene of Archaea, the methanogenic orders (Methanomicrobiales, Methanobacteriales, and Methanococcales), and the families Methanosaetaceae and Methanosarcinaceae. We also used qPCR to target the 16S rRNA gene of Bacteria [39] and the highly conserved formyl tetrahydrofolate synthase (FTHFS) gene in homoacetogens by performing SYBR green assays, using methods described previously [38].

### 2.7. Statistical Analysis

Using the Statistical Package for the Social Sciences (SPSS) software, we performed Pearson's parametric correlation among variables of interest: methane production, total N and NH_3_-N concentrations, and key fermenters, syntrophs, and hydrogenotrophic and acetoclastic methanogens identified through sequencing analysis. These variables were picked based on the hypothesis that NH_3_-N would have an effect on microbial community structure, specifically on methanogens, and correction for multiple comparisons was not performed. *P* < 0.05 was accepted as significant.

## 3. Results and Discussion

### 3.1. COD Was Converted into Methane during Bioreactor Operation despite NH_3_-N > 2000 mg/L

**Figure 1:**
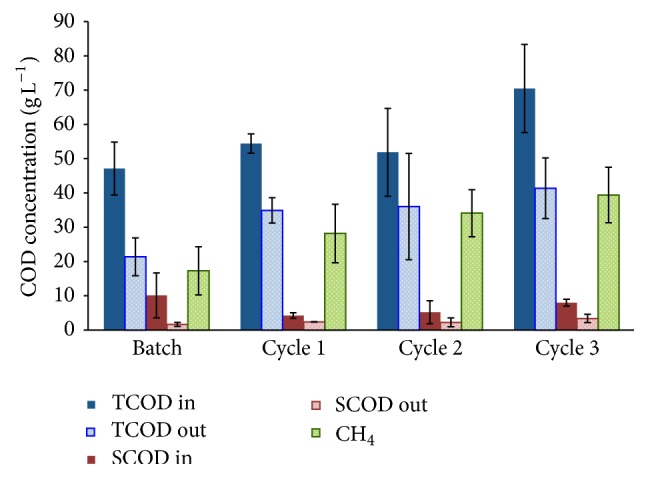
Total COD (TCOD) and soluble COD (SCOD) in the influent and effluent of the reactor and the COD content of the total methane (CH_4_) produced during each operating phase (batch and 3 cycles of semicontinuous operation). The data are averages with standard deviations of three or more measurements.

**Figure 2:**
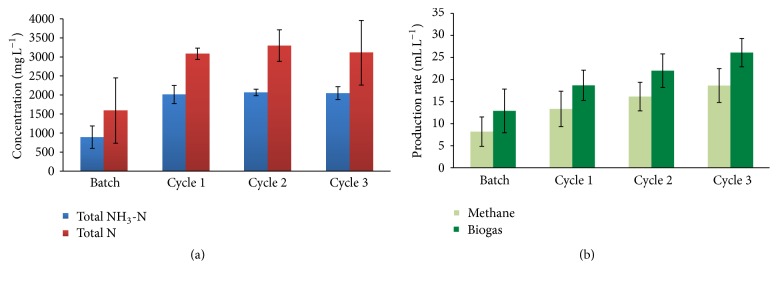
(a) Total nitrogen and NH_3_-N concentrations and (b) methane and total biogas production rates during batch and semicontinuous operation. The data are averages with standard deviations of three or more measurements during each operating phase.

We monitored total and soluble COD, NH_3_-N and total N, and methane and biogas production rates at regular intervals during the startup phase of the methanogenic reactor treating swine waste. The results are summarized in Figures 1 and 2 and S1, in Supplementary Material available online at http://dx.doi.org/10.1155/2016/4089684; Table S1 documents good COD mass-balance closure at four sampling times. Based on COD removed as CH_4_, the performance of the reactor approached a pseudo-steady state in cycle 1 of semicontinuous operation, with approximately 52% conversion. CH_4_ was 70% ± 16% of the biogas, and H_2_ concentrations were below detectable levels (<0.5% v/v) throughout the experiment. During semicontinuous operation, effluent soluble COD (which is composed of relatively small, biodegradable molecules [39]) represented only 4.5% ± 0.3% of the influent TCOD or ≤2.6 ± 0.6 g COD L^−1^. This low SCOD concentration implies that short-chain fatty acids (including propionate, butyrate, and acetate), which mainly comprise the SCOD, did not accumulate because they were consumed by microbial metabolism leading to methane production and biomass synthesis.

Figure 2(a) shows NH_3_-N increased from 890 ± 295 mg NH_3_-N L^−1^ during batch operation to 2040 ± 30 mg NH_3_-N L^−1^ during semicontinuous operation. The concentration of soluble total N (the sum of NH_3_-N and organic N) paralleled that of NH_3_-N and was about 50% higher than total NH_3_-N. This increasing release of organic N and NH_3_-N indicates that hydrolysis and fermentation of the protein fraction of the animal wastes increased after startup and stabilized in cycle 2.  Figure 2(b) shows that methanogenesis continued to increase into cycle 3, even though hydrolysis and fermentation of protein stabilized. Higher methane generation was possible in cycle 3 because the input TCOD increased with the batch of swine waste used in that cycle; the input N did not increase in parallel with input TCOD because the feed collected swine manure was not uniform throughout the experimental period.

### 3.2. The Diversity of Archaea and Bacteria Decreased with Increasing NH_3_-N Concentration

Table 2 summarizes the coefficients of the two metrics used to analyze diversity within the samples. PD-whole-tree, which is based on the phylogenetic tree, uses the branch lengths as a measure of diversity; the observed-species metric counts all unique OTUs in the sample [40]. Both metrics had similar decreasing trends for Archaea and Bacteria over time as the NH_3_-N concentration rose from 684 mg NH_3_-N L^−1^ at the startup of the reactor to 890 ± 295 mg NH_3_-N L^−1^ in batch operation and to 2040 ± 30 mg NH_3_-N L^−1^ for continuous operation. These significant decreases suggest selective enrichment of microorganisms tolerant to NH_3_-N.

### 3.3. Hydrogenotrophic and Acetoclastic Methanogens Were Abundant at ~2000 mg NH_3_-N L^−1^

**Figure 3:**
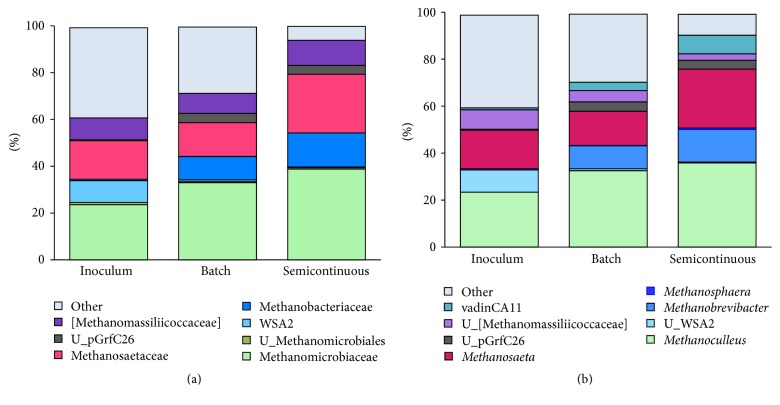
Archaeal distributions. (a) Phylotypes at the family level. (b) Phylotypes at the genera level. Shades of green are for Methanomicrobiales (hydrogenotrophic methanogens), blue for Methanobacteriales (hydrogenotrophic methanogens), red for Methanosarcinales (acetoclastic methanogens), and black for pGrfC26. The total NH_3_-N concentration at the time of the batch sampling was 1140 mg/L. Semicontinuous operation is an average of two samples at ~2000 mg NH_3_-N/L. “U_” stands for unidentified microorganism within the taxonomic classification.

We analyzed the Archaeal microbial community during reactor startup using high-throughput sequencing in order to evaluate how well acetoclastic methanogens (grouped under Methanosarcinales) or hydrogenotrophic methanogens (grouped under Methanobacteriales, Methanomicrobiales, Methanococcales, and the E2 order) were tolerant to NH_3_-N concentrations of ~2000 mg L^−1^.  Figure 3 compares the Archaeal communities in the inoculum with the communities after batch and semicontinuous operation. Phylotypes within the phylum Euryarchaeota (i.e., Methanomicrobiales, Methanobacteriales, E2 group, and Methanosarcinales) and those of the order pGrfc26 (within Crenarchaeota) [41] increased during the semicontinuous operation at 2040 ± 30 mg NH_3_-N L^−1^, while the unidentified phylotypes decreased from 38.6 to 1.5%. These results confirm enrichment during the gradual acclimation to high and increasing total-ammonium concentrations.

The relative abundance of hydrogenotrophic and acetoclastic methanogens increased over time. This agrees with increasing methane production, low effluent SCOD (including acetate in the measurement), and no detection of H_2_ during semicontinuous operation.  Figure 3(b) shows 8 different genera of hydrogenotrophic methanogens identified, including* Methanoculleus*,* Methanogenium, *and* Methanobrevibacter*. Although the genus* Methanosaeta* was the sole acetoclastic methanogen identified by high-throughput sequencing, its relative abundance increased from 14% in batch operation to 25% during semicontinuous operation. This increase in relative abundance could be due to an increase in the absolute abundance of* Methanosaeta* or a decrease in the abundance of other phenotypes. qPCR results (summarized in Figure S2) are consistent with Figure 3 and suggest that Methanomicrobiales were the most abundant methanogens followed by Methanosaetaceae and lastly by Methanobacteriales.

It is likely that the first 35 d of batch operation, with <1100 mg N-NH_3_ L^−1^, provided an acclimation period for acetoclastic methanogens, and ~2000 mg NH_3_-N L^−1^ was not high enough to inhibit* Methanosaeta*, which were selected by the third cycle, relative to other Archaea. This corresponds to the finding by Schnürer and Nordberg that it took 3000 mg NH_3_-N L^−1^ to inhibit NH_3_-N-acclimated* Methanosaeta* spp. [18] and that this family was not detected in anaerobic digesters treating chicken wastes with above 3400 mg NH_3_-N L^−1^ [42]. However, a recent study reported that NH_3_-N-acclimated Methanosaetaceae spp. were the most abundant acetoclastic methanogens identified in laboratory-scale anaerobic digesters operated at increasing NH_3_-N concentrations of up to 4000 mg L^−1^ [43].

### 3.4. Acetogens Played a Key Role in Methane Production at ~2000 mg NH_3_-N L^−1^

**Figure 4:**
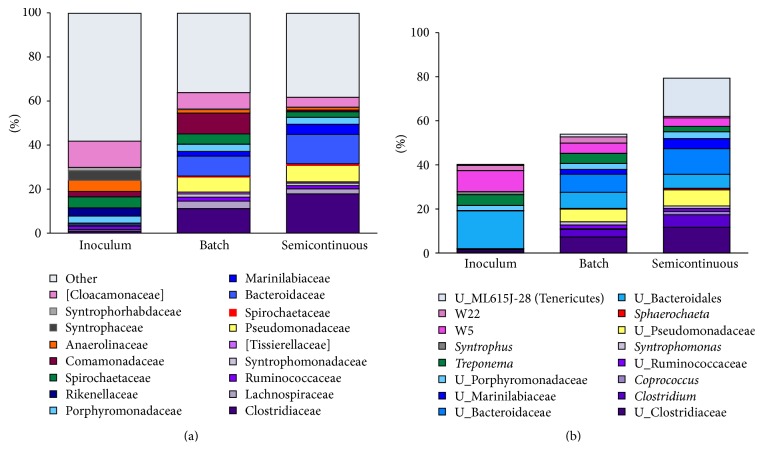
Bacterial distributions. (a) Phylotypes at the family level. (b) Most abundant phylotypes at the genera level. Similar colors (purple, yellow, black, blue, green, gray, and pink) indicate that the families are in the same order. The total NH_3_-N concentration at the time of the batch sampling was 1140 mg/L. Semicontinuous operation is an average of two samples at ~2000 mg NH_3_-N/L. “U_” stands for unidentified microorganism within the taxonomic classification.

The most abundant Bacterial phylotypes at the family and genus levels are summarized in Figure 4. At the genus level, phylotypes representative of producers of key short-chain fatty-acid (e.g., acetate, propionate, and butyrate) were enriched during exposure to high NH_3_-N. These Bacteria are summarized in panel (b). The most abundant phylotypes were (i)* Coprococcus*, a butyrate- and acetate-producer within the Lachnospiraceae family [44], (ii)* Sphaerochaeta* (within the Spirochaetaceae family), an acetate-, formate-, and ethanol-producer [45], (iii)* Treponema*, acetogenic microorganisms within the Spirochaetaceae family, and (iv) unidentified phylotypes in the Bacteroidales and Clostridiales orders (represented in purple and blue, resp.), known to harbor microorganisms capable of fermenting carbohydrates and proteins to short-chain fatty-acids [46–48].

**Figure 5:**
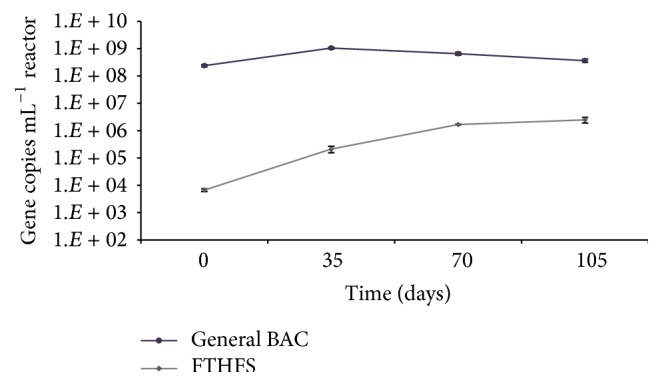
Gene copies for General Bacteria (BAC) and the FTHFS gene (marker of homoacetogens) over the duration of operation of the semicontinuous reactor.

To elaborate on the acetogens, we used qPCR to quantify the highly conserved formyl tetrahydrofolate synthetase (FTHFS) gene of homoacetogens [49, 50] and reverse-acetogens (sometimes called “syntrophic acetate oxidizers”) [6, 21].  Figure 5 shows a steady increase of the FTHFS gene; quantified FTHFS genes increased about two orders of magnitude from the beginning of the experiment to day 105 despite relative constant numbers of Bacterial 16S rRNA genes. This indicates that there was not only an increase in homoacetogenic Bacteria but also a relative increase in their proportion of the Bacterial community, revealing an enrichment of homoacetogens with increasing NH_3_-N concentrations.

Low SCOD in the effluent, no detection of H_2_, and the high amount of acetoclastic and hydrogenotrophic methanogens mean that acetate and H_2_ were efficiently scavenged to produce CH_4_. The presence of homoacetogens means that the sink for acetate during semicontinuous operation could have been either one of the methanogenic pathways or both. Homoacetogens could either have been doing forward acetogenesis, in which case acetoclastic methanogens scavenge acetate, or have been doing reverse acetogenesis, in which hydrogenotrophic methanogens feed on H_2_ and CO_2_ generated from acetate.

Syntrophic acetate-utilizers were represented by phylotypes associated with the genus* Clostridium* within the Clostridiaceae family. Their relative abundance increased from 0.72% in the inoculum to 3.5 and 5.6% during batch and semicontinuous operation, respectively. Some strains (including* C. ultunense *[51] and strains similar to* C. botulinum*,* C. sticklandii*, and* C. beijerinckii* [52]) have been reported to perform reverse acetogenesis in methanogenic communities [51, 52]. With high NH_3_-N concentration, these acetate-utilizers have been commonly found in syntrophy with hydrogenotrophic methanogens such as* Methanoculleus* [13, 53], the most abundant methanogen identified in our reactor. Thus, it is possible that several* Clostridium* spp. in our reactor contributed to methane production through reverse acetogenesis coupled with hydrogenotrophic methanogenesis.

### 3.5. Syntrophic Fatty-Acid Fermenters Thrived at ~2000 mg NH_3_-N L^−1^

In addition to syntrophic acetate-utilizers among the Clostridia, propionate- and butyrate-fermenters that grow in syntrophy with hydrogenotrophic methanogens also were detected at relative abundances between 1.2 and 4.5% during semicontinuous operation. The detected phylotypes at the genus level were* Syntrophomonas* and W22 and W5.* Syntrophomonas* (within the Syntrophomonadaceae) ferment butyrate to acetate and H_2_ in syntrophic association with hydrogenotrophic methanogens and sulfate-reducers [22, 54].* Cloacamonas*, a representative genus of Cloacamonaceae, obtains its energy from the fermentation of amino acids and can ferment propionate to acetate, H_2_, and CO_2_ in syntrophy with H_2_ and acetate consumers [55, 56]. This syntroph has been named* Candidatus Cloacamonas acidaminovorans*, and although it has not been cultivated, its genome has been reconstructed by metagenomics [55]. Comparing the sequences (300 bp) associated with the W22 and W5 genera to available sequences (NCBI, BLAST) reveals that these genera share up to 96% similarity with* Candidatus C. acidaminovorans*. Therefore, it is possible that Cloacamonaceae and Syntrophomonadaceae contributed to methane production with 2040 ± 30 mg NH_3_-N L^−1^ by fermenting butyrate and propionate to H_2_, CO_2_, and acetate in syntrophy with hydrogenotrophic and acetoclastic methanogens.

### 3.6. Correlation Analysis Supports Syntrophies between Acetoclastic Methanogens with Acetogens and Hydrogenotrophic Methanogens with Syntrophic Fatty-Acid Fermenters

**Figure 6:**
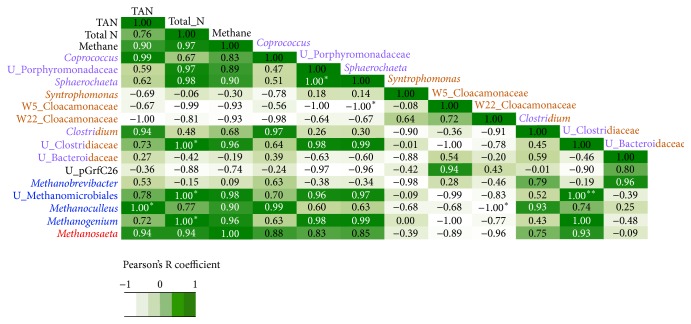
Parametric correlation (Pearson's R coefficient) of methane production, total NH_3_-N (TAN) concentration, and key Bacteria and Archaea identified during operation of the anaerobic reactor treating swine waste. Phylotypes in purple, orange, blue, and red represent fermenters, syntrophs, and hydrogenotrophic and acetoclastic methanogens, respectively. Phylotypes that are most similar to fermenters and syntrophs are indicated in a combination of purple and orange colors.* Note*.  ^*∗*^Correlation is significant at the 0.05 level (2-tailed). ^*∗∗*^Correlation is significant at the 0.01 level (2-tailed). The statistical analysis was not corrected for multiple comparisons.

In order to understand the effect of NH_3_-N and total N concentrations on methane production and the microbial community structure, we calculated Pearson's R coefficient among methane production rates, NH_3_-N and total N concentrations, and fermenters, syntrophs, and hydrogenotrophic and acetoclastic methanogens identified at four time points during operation of the anaerobic reactor treating swine waste. The results of the parametric correlation analysis are summarized in Figure 6. Hydrogenotrophic methanogens (*Methanoculleus*,* Methanogenium*,* Methanobrevibacter, *and an unidentified genus within Methanomicrobiales) and acetoclastic* Methanosaeta* were positively correlated with total N and NH_3_-N concentrations (in some cases, correlations were significant at the 0.05 level). These positive correlations suggest that hydrogenotrophic and acetoclastic methanogens thrived with increasing NH_3_-N concentration up to 2040 ± 30 mg NH_3_-N L^−1^ when the pH was 6.9–7.6 and the HRT was 35 d. Moreover, acetogens/fermenters* Coprococcus*,* Sphaerochaeta*, and an unidentified genus within Porphyromonadaceae were positively correlated with NH_3_-N and almost all methanogens and, consequently, correlation with methane production was positive. These positive correlations underscore the important role of acetogens for methane production at high NH_3_-N.

Unidentified Clostridiaceae, which comprises several short-chain fatty-acid producers, and* Clostridium *had positive correlations with acetoclastic* Methanosaeta*, and this supports acetate generation by acetogens and homoacetogens. However, unidentified Clostridiaceae also showed a positive correlation with unidentified hydrogenotrophic Methanomicrobiales and with hydrogenotrophs* Methanogenium *and* Methanoculleus. *This supports that homoacetogens among the Clostridiaceae were possibly carrying out reverse acetogenesis. Thus, parametric analysis supports an important role for homoacetogens, but it cannot determine whether they were performing forward or reverse acetogenesis.

## 4. Conclusions

**Figure 7:**
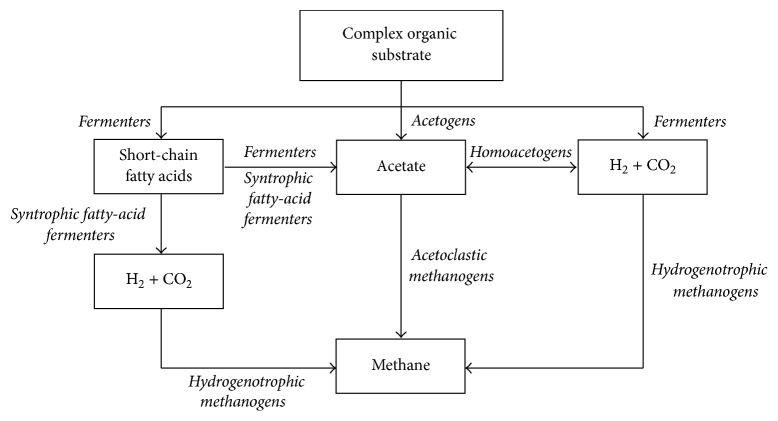
Proposed key anaerobic food-web reactions occurring at ammonia-N concentrations of ~2000 NH_3_-N mg L^−1^.

Successful operation of an anaerobic reactor treating swine manure proved that Bacterial and Archaeal communities could acclimate to a steady increase in total NH_3_-N concentration up to 2040 ± 30 mg NH_3_-N L^−1^. Both communities became less diverse over time. NH_3_-N tolerant phylotypes that were enriched include (1) acetoclastic methanogens (*Methanosaeta*); (2) Clostridia known to do forward and reverse acetogenesis (*Clostridium* and Clostridiaceae spp.); (3) fatty-acid producers (*Coprococcus* and* Sphaerochaeta*); (4) hydrogenotrophic methanogens (*Methanoculleus*,* Methanobrevibacter*, and* Methanogenium*); and (5) syntrophic fatty-acid fermenters (*Syntrophomonas*,* Clostridium*, Clostridiaceae spp., and possibly Cloacamonaceae species). Our results suggest that the gradual increase in the NH_3_-N concentration led to a microbial community acclimated to the high total NH_3_ concentrations associated with anaerobic digestion of animal wastes. As summarized in Figure 7, acetoclastic and hydrogenotrophic methanogens could coexist in the presence of NH_3_-N concentrations ~2000 mg L^−1^ by establishing syntrophic relationships with propionate and butyrate-fermenters, as well as homoacetogens able to carry out forward and reverse acetogenesis.

## Supplementary Material

Figure S1. Methane and total biogas production rates in the semi-continuous reactor through 140 days.

## Figures and Tables

**Table 1:** Stoichiometry and thermodynamics of syntrophic acetate, propionate, and butyrate fermentation.

Acetate fermentation	(1a) CH_3_COO^−^ + H^+^ + 2H_2_O → 2CO_2_ + 4H_2_	Δ*G*°′ = +55.0
Propionate fermentation	(1b) C_3_H_5_OO^−^ + 2H_2_O → CH_3_COO^−^ + CO_2_ + 3H_2_	Δ*G*°′ = +76.0
Butyrate fermentation	(1c) C_4_H_7_OO^−^ + 2H_2_O → 2CH_3_COO^−^ + H^+^ + 2H_2_	Δ*G*°′ = +48.3
Hydrogen oxidation	(2) 4H_2_ + CO_2_→ CH_4_ + 2H_2_O	Δ*G*°′ = −130.8
(1a) + (2)	(3) CH_3_COO^−^ + H^+^→ CO_2_ + CH_4_	Δ*G*°′ = −75.8
(1b) + (2)	(4) C_3_H_5_OO^−^ + (1/2)H_2_O → CH_3_OO^−^ + (3/4)CH_4_ + (1/4)CO_2_	Δ*G*°′ = −28.0
(1c) + (2)	(5) C_4_H_7_OO^−^ + 2H_2_ + CO_2_→ 2CH_3_COO^−^ + H^+^ + CH_4_	Δ*G*°′ = −42.6

The standard free enthalpies of formation (Δ*G*°′) are reported in kJ reaction^−1^ at 1 M, pH 7, and 25°C.

**Table 2:** Diversity of each sample at different time points during the startup phase of the methanogenic reactor treating swine waste. At the highest NH_3_-N concentration, samples species diversity both for Archaea and for Bacteria was lowest.

Sample	NH_3_-N (mg/L)	Bacteria		Archaea	
		PD-whole-tree	Observed-species	PD-whole-tree	Observed-species
Inoculum	N/A	67 ± 0.3	1512 ± 10	24 ± 0.2	375 ± 6
Batch	<1100	62 ± 0.3	1419 ± 7	25 ± 0.4	474 ± 10
^*∗*^Semicontinuous	~2000	52 ± 0.4	1173 ± 12	21 ± 0.1	361 ± 2

^*∗*^The indices corresponding to semicontinuous operation are averages of the indices for samples taken on days 70 and 105.
