# Supplementary material for: Archaea and Bacteria Acclimate to High Total Ammonia in a Methanogenic Reactor Treating Swine Waste
Source: Archaea. 2016 Sep 20;2016:4089684. doi: 10.1155/2016/4089684 (PMC5048046; doi:10.1155/2016/4089684)
Supplement: Supplementary file 1 — Figure S1. Methane and total biogas production rates in the semi-continuous reactor through 140 days. [file 4089684.f1.pdf]

Archaea and Bacteria acclimate to high total ammonia in a methanogenic reactor treating swine waste

Sofia Esquivel-Elizondo[1, 2§], Prathap Parameswaran[3§], Anca G. Delgado[1], Juan Maldonado[1], Bruce E. Rittmann[1,2], and Rosa Krajmalnik-Brown[1,2#]

[1]Swette Center for Environmental Biotechnology, The Biodesign Institute, Arizona State University, P.O. Box 875701, Tempe, AZ 85287-5701, USA

[2]School of Sustainable Engineering and the Built Environment, Arizona State University, Tempe, AZ

[3]Department of Civil Engineering, Kansas State University, 2118 Fiedler Hall, Manhattan, KS 66506

[§]Authors contributed equally to this work

[#]Correspondence should be addressed to Rosa Krajmalnik-Brown; dr.rosy@asu.edu

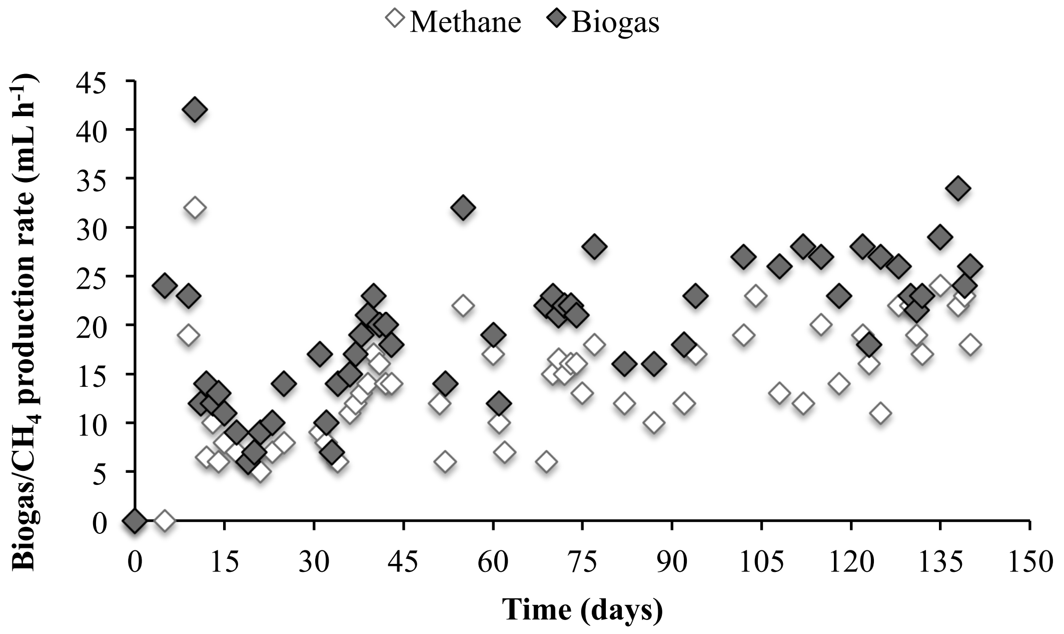

Figure S1. Methane and total biogas production rates in the semi-continuous reactor through 140 days.

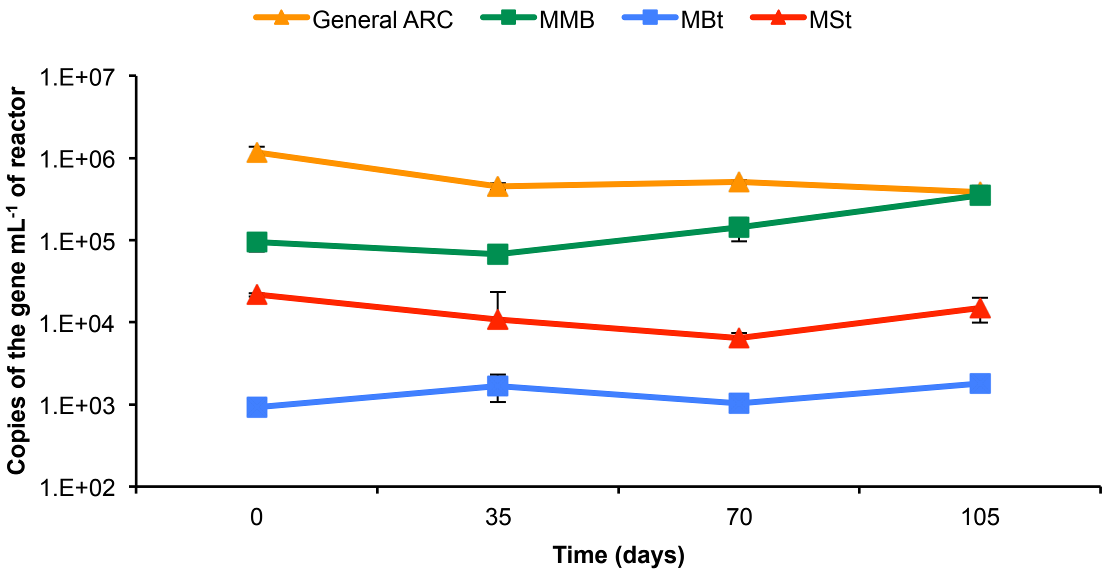

Figure S2. Gene copies per mL of reactor for General Archaea (ARC), *Methanomicrobiales* (MMB), *Methanobacteriales* (MBt), and *Methanosaetaceae* (MSt) over the duration of operation of the semi-continuous reactor.

Table S1. Summary COD mass balances at different phases during the semi-continuous bioreactor operation. The % error indicates the deviation in the closure of the COD mass balance. The times for the analysis were selected based on steady methane production.

Time (day, cycle)	Soluble COD_{in} (g)	Total COD_{in} (g)	Soluble COD_{out} (g)	Total COD_{out} (g)	COD_{out} as CH_4 (g)	$TCOD_{in}$- [Total COD_{out} + COD_{out} as CH_4] (g)	% error in COD balance
53, 1	0.13	1.97	0.09	1.14	0.60	− 0.23	12
94, 2	0.64	2.51	0.14	1.74	0.89	− 0.12	4.8
122, 3	0.33	1.94	0.13	1.13	0.89	− 0.08	− 4.1
138, 3	0.31	2.66	0.13	1.63	0.11	− 0.02	− 0.75
